# In Vitro Modeling of Bradykinin-Mediated Angioedema States

**DOI:** 10.3390/ph13090201

**Published:** 2020-08-19

**Authors:** François Marceau, Hélène Bachelard, Xavier Charest-Morin, Jacques Hébert, Georges E. Rivard

**Affiliations:** 1 Centre de Recherche du CHU de Québec-Université Laval, Québec, QC G1V 4G2, Canada; helene.bachelard@crchudequebec.ulaval.ca (H.B.); xav_c_moi@hotmail.com (X.C.-M.); 2 Service D’allergie, CHU de Québec-Université Laval, Québec, QC G1V 4G2, Canada; hebert.j@videotron.ca; 3 CHU Sainte-Justine, Université de Montréal, Montréal, QC H3T 1C5, Canada; georges-etienne.rivard.hsj@ssss.gouv.qc.ca

**Keywords:** hereditary angioedema, bradykinin, acquired angioedema, kallikrein–kinin system, analytical techniques for kinins

## Abstract

Kinins (peptides related to bradykinin, BK) are formed from circulating substrates, the kininogens, by the action of two proteases, the kallikreins. The only clinical application of a BK receptor ligand, the B_2_ receptor antagonist icatibant, is the treatment of the rare hereditary angioedema (HAE) caused by the deficiency of C1-esterase inhibitor (C1-INH). Less common forms of HAE (genetic variants of factor XII, plasminogen, kininogen) are presumably mediated by increased BK formation. Acquired forms of BK-mediated angioedema, such as that associated with angiotensin-I converting enzyme (ACE) inhibition, are also known. Antibody-based analytical techniques are briefly reviewed, and support that kinins are extremely short-lived, prominently cleared by ACE. Despite evidence of continuous activation of the kallikrein–kinin system in HAE, patients are not symptomatic most of the time and their blood or plasma obtained during remission does not generate excessive immunoreactive BK (iBK), suggesting effective homeostatic mechanisms. HAE-C1-INH and HAE-FXII plasmas are both hyperresponsive to fibrinolysis activation. On another hand, we suggested a role for the alternate tissue kallikrein–kinin system in patients with a plasminogen mutation. The role of the BK B_1_ receptor is still uncertain in angioedema states. iBK profiles under in vitro stimulation provide fresh insight into the physiopathology of angioedema.

## 1. The Kallikrein–Kinin System

**Figure 1:**
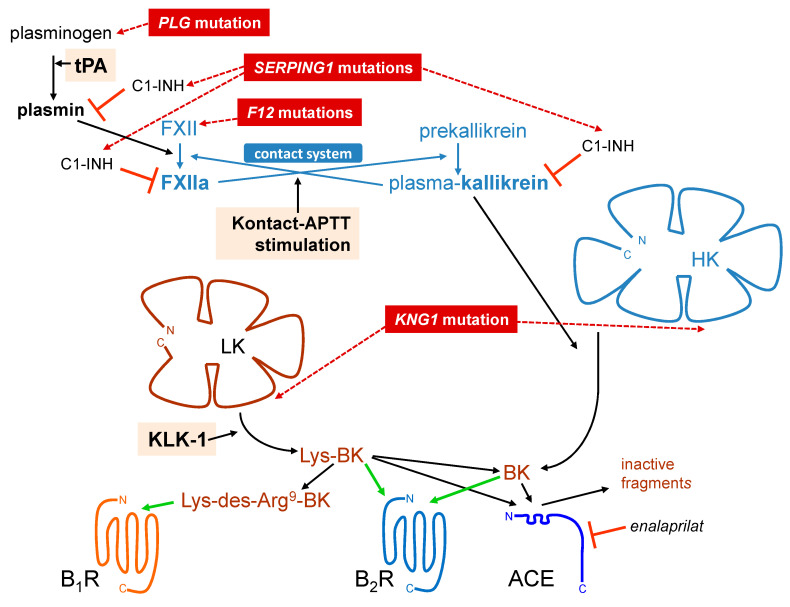
Schematic representation of the kallikrein-kinin system, featuring the 2 validated pathways of kinin generation, that of plasma kallikrein (part of the contact system) releasing bradykinin (BK) from high molecular weight kininogen (HK) and that mediated by secreted tissue kallikrein (KLK-1), generating Lys-BK mainly from low molecular weight kininogen (LK). Some of the known mutant genes that cause HAE-nC1-INH (dark red background with dotted arrows pointing to the corresponding mutant proteins) and the standardized stimuli applied in vitro to trigger kinin formation (pale orange background) are also indicated. Elements of the contact system are represented in light blue. Black and blue arrows represent biochemical reactions. Green arrows indicate an agonist effect on a receptor. “⊥” Inhibition of a protease or peptidase. See Table 1 for abbreviations.

The present paper reviews recent literature, contains some original results, and aims to illustrate the role of the analytical biochemistry of BK-related kinins in the understanding of angioedema (AE) mediated by kinins. The kallikrein –kinin system is made of many molecular participants present or secreted in blood plasma or present at the vascular endothelial surface [1,2,3] (Figure 1). There are two different precursors of kinins, low and high molecular weight kininogens (LK, HK; abbreviations are defined in Table 1). Those proteins are encoded by alternative splicing of a single gene (*KNG1*) and produced by the liver. They share a common sequence in most domains, including the BK sequence embedded in domain 4 [1]. Tissue kallikrein (KLK-1) is produced as a zymogen by many tissues like the salivary glands and pancreas, but also by renal cells and vascular endothelial cells [4]. Human KLK-1 is member of a large family of 15 serine proteases. LK is the most abundant kininogen and KLK-1 releases Lys-BK (or kallidin) mostly from it, but also from HK [3]. Lys-BK is a direct agonist of the BK B_2_ receptor [5] (Figure 1). Arginine carboxypeptidases can remove the C-terminal Arg residue from Lys-BK to produce Lys-des-Arg^9^-BK (des-Arg^10^-kallidin), the only subnanomolar affinity agonist of the human form of the B_1_ receptor [5] (Figure 1). A fact that is often overlooked is that des-Arg^9^-BK, a very potent agonist of the B_1_ receptor in rodent species, has a very weak affinity for the human B_1_ receptor; thus, this receptor subtype can be said to be compartmentalized with one of the two kinin-generating system, tissue kallikrein (KLK-1). The release and maturation of KLK-1 are not well understood, but they are known to have roles in different tissues, namely intrarenal hemodynamics, flow-dependent vasodilation and other compensatory mechanisms. They also have a role in inflammation, notably in the airways and gut [4,6,7,8].

The other kinin-generating system revolves around plasma kallikrein and its associated components in the contact system (Figure 1, represented in light blue). HK is bound to the zymogen prekallikrein and to coagulation factor XI (FXI) via specific domains. This complex may be loosely bound to sites present on endothelial cells [1] but has a natural affinity for negatively charged surfaces, such as the denuded basal membrane of damaged endothelium, sodium urate crystals, platelet delta granule insoluble polyphosphates (see below), and in the laboratory, glass, kaolin, and dextran sulfate. Soluble factor XII (FXII) also has an affinity to such abnormal surfaces: it and prekallikrein mutually activate each other into active proteases, factor XIIa (FXIIa) and plasma kallikrein, respectively. The latter releases the vasoactive mediator BK from HK and FXIIa initiates the intrinsic coagulation pathway via FXI activation and subsequent reactions. BK is a high affinity agonist of the B_2_ receptor, found in endothelial cells, sensory neurons, some epithelia and other cell types [5]. The most immediate vascular effect of kinins is vasodilation, mediated by the endothelial production of nitric oxide and prostanoids via calcium signaling, and disruption of the permeability barrier due to a contraction of the endothelial cells and simultaneous Tyr-phosphorylation of vascular endothelial cadherin (VE-cadherin) followed by its breakdown at the intercellular adherens junctions [9]. These effects are particularly relevant to AE states. The fibrinolytic system, composed of plasminogen and its upstream activating proteases, such as tissue plasminogen activator, are increasingly discussed in AE physiopathology. Plasmin recruits the contact system via the cleavage of FXII [10] (Figure 1).

The kallikreins are endogenously controlled by circulating serine protease inhibitors (serpins). C1 esterase inhibitor (C1-INH; serpin G1) abates the effect of plasma kallikrein, but also of FXII, plasmin and complement component C1 (Figure 1). Tissue kallikrein is inhibited by endogenous kallistatin (serpin A4) [11]. Whole citrated blood was used in our initial work with iBK measurements to verify previous claims of BK generation by the activation of neutrophil leukocytes or platelets, via the secreted protease PR3 or NETosis in the first case, or secretory products such as polyphosphate in the second [12]. Despite ample evidence of the activation of neutrophils and platelets following relevant in vitro stimulation, no measurable production of iBK was evidenced. This could be explained by the presence of the multiple endogenous protease inhibitors of plasma [12].

## 2. Bradykinin (BK)-Mediated Angioedema States

BK-mediated AE is a rare acquired or hereditary condition involving localized edema of subcutaneous and submucosal tissues. This group of disorders may be life-threatening (e.g., via suffocation), very painful and incapacitating. The mechanism is mast-cell independent and differential diagnosis must exclude various allergic or urticarial conditions that are much more frequent [13]. The most common cause of acquired AE is drug-induced, in the context of treatment with angiotensin-I converting enzyme (ACE) inhibitors for cardiovascular conditions [14,15]. The basis of this disorder may be an insufficient clearance of BK, as ACE is by far the major peptidase that inactivates kinins in blood/plasma/serum and in vivo [16,17,18]. However, what triggers kinin formation in affected patients is unclear [19]. Therapeutic fibrinolysis, with recombinant tissue plasminogen activator (tPA) or comparable plasmin activators, is associated with AE or anaphylactoid reactions. BK production initiated by plasmin-mediated FXII activation may be involved in these cases [20,21]. Non-hereditary forms of BK-mediated AE include those associated with low levels of C1-INH due to autoantibodies or overconsumption (lymphoproliferative disease) [22].

Hereditary angioedema (HAE), an autosomal dominant group of disorders, involves several gene variants that are proven or postulated to be permissive for kinin production (Figure 1) [22]. Many variants of the *SERPING1* gene encoding C1-INH with impaired production (type I HAE-C1-INH) or dysfunctional (type II HAE-C1-INH) forms of this serpin, cause the most common form of HAE. Rarer forms of HAE with normal C1-INH levels are caused by mutation of genes encoding coagulation FXII (*F12*), plasminogen (*PLG*), or of kininogens (*KNG1*) [22]. Some variants of FXII that are associated with HAE-FXII introduce new sites of cleavage by plasmin that accelerate cleavage by this protease, a plausible basis for a gain of function [23]. HAE associated with variants of the angiopoietin 1 gene (*ANGPT1*) suggests a different general mechanism of the disease where the endothelial permeability barrier is primarily affected [22,24]. However, for many patients, no mutation or abnormalities have as yet found and they may differ in several aspects, including gender distribution, genetics, symptoms, and estrogen impact.

Animal models of HAE-C1-INH do not fully recapitulate the human disease. Both homozygous and heterozygous C1-INH-deficient mice failed to exhibit edema attacks, but nevertheless had an increased basal microvascular permeability mediated by plasma kallikrein and the BK B_2_ receptor [25]. It has been argued that endothelium-specific overexpression of B_2_ receptors in rats, under the promoter of VE-cadherin, resulted in a more representative model because localized BK-dependent plasma extravasation could be triggered by the application of an irritant and spontaneous abdominal edema was occasionally observed [26].

Ultimately, various forms of AE attacks involve the excessive stimulation of the endothelial BK B_2_ receptor and ensuing increased microvascular permeability. The only FDA-approved clinical use of a BK receptor ligand is that of icatibant, an injectable peptide antagonist of the B_2_ receptor, to abort attacks of HAE-C1-INH. C1-INH replenishment and various strategies to inhibit plasma kallikrein, FXII or fibrinolysis have also been or are being developed [27]. An orally bioavailable small molecule antagonist of the BK B_2_ receptor is also at an early stage of development [28].

## 3. Antibody-Based Analytical Techniques for Kinins: Importance and Pitfalls

**Figure 2:**
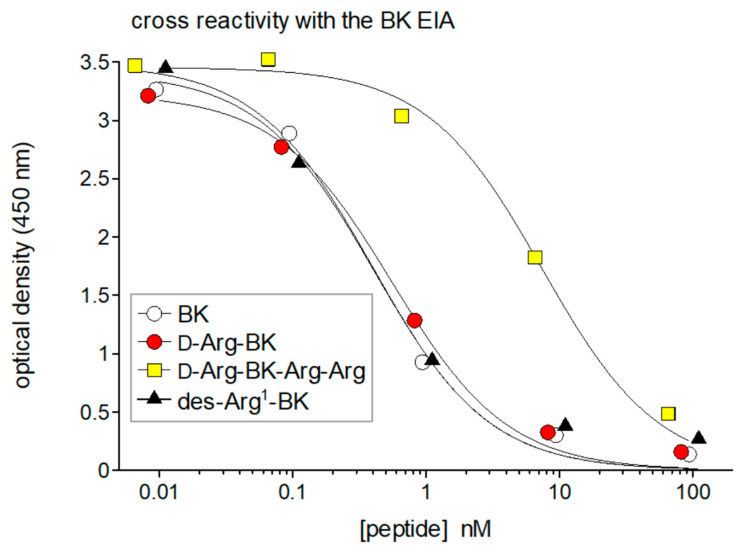
Cross-reactivity of the BK EIA with selected kinin sequences. The inactive metabolite des-Arg^1^-BK (= BK fragment 2–9) exhibits full cross-reactivity vs. BK. N-terminal extended sequences, like Arg-BK (shown here), or Lys-BK are also fully reactive. Truncated sequences at the C-terminus, like the optimal B_1_ receptor agonist Lys-des-Arg^9^-BK, do not cross react at all [12]. An extended sequence like Arg-BK-Arg-Arg, an inactive prodrug releasing active kinins via the action of carboxypeptidases [31], has a partial reactivity, showing the importance of a free COOH terminus. Reproduced in part from [12].

BK and related peptides are formed locally close to their site of action (autacoids). They are extremely unstable and prone to artefactual formation when blood or tissues are sampled (for instance, following contact of blood with glass). Therefore, measuring kinins in venous blood is not really informative [3], contrasting with many useful peptide hormone dosages applied in endocrinology. A further challenge in concentration determination is that the BK sequence is embedded in that of precursor kininogens, with necessary separation if an antibody-based detection system is considered. Blais et al. [3] have reviewed these pitfalls and discussed extraction procedures and recovery in the context of the currently applied immunoassays. Professor A. Adam and co-workers developed separate competitive chemiluminescence enzyme immunoassays (EIAs) for BK and des-Arg^9^-BK based on digoxygenin-conjugated peptides [29,30]. Now several commercially available kits are being used, such as that from Phoenix Pharmaceuticals for BK (96-well plate format), and these have the advantage of world-wide reproducibility. The sensitivity of EIAs is excellent. Typical of peptide tracers with N-terminal extensions (digoxygenin, biotin), the polyclonal anti-BK antibodies react with only a few C-terminal amino acids in the kinin sequences; thus N-terminally extended sequences like Lys-BK and Ile-Ser-BK fully cross-react in the BK EIA, but not the C-terminally truncated des-Arg^9^-BK [29]. Similarly, Phoenix Pharmaceuticals states that its BK EIA fully cross-reacts with Lys-BK or biotinyl-BK, but not with des-Arg^9^-BK. Using this assay, we observed that the N-terminally extended sequence protected from aminopeptidases, D-Arg-BK, is also equipotent with BK in the EIA, but that the C-terminally extended peptide D-Arg-BK-Arg-Arg loses much affinity, consistent with the antibody preference for an intact C-terminal BK sequence (Figure 2).

A particular concern is the full EIA reactivity of the BK fragment des-Arg^1^-BK (Figure 2), and possibly of other N-terminally truncated BK fragment, again supporting that the antibodies recognize only a limited C-terminal sequence. Des-Arg^1^-BK is inactive as an agonist of either the B_1_ or B_2_ receptors [32] (Figure 2). Thus, the EIA signal may be contaminated with the inert metabolite generated by aminopeptidase P, a significant peptidase that inactivates BK [17]. One way out is to couple the EIA quantification with chromatographic separation of kinins, such as HPLC [29]. Another approach is to verify the presence of BK-like activity in extracts using bioassays. Thus, Adam et al. [33] used ERK1/2 signaling in cells expressing rabbit B_2_ receptors to confirm iBK in extracts of plasma stimulated with contaminated heparin. Later, the accumulation of c-Fos in HEK 293a cells expressing the human B_2_ receptor was successfully used as a discriminative bioassay to confirm measurements of iBK in extracts of human blood or plasma [12,18]. Lys-BK and BK are approximately equipotent as agonists of the B_2_ receptor, so they add up in EIA results as “iBK” and in bioassay results as well, if either one or both are present in a given extract. Similarly, the published des-Arg^9^-BK EIA, based on the tracer digoxygenin-des-Arg^9^-BK, was equally reactive with Lys-des-Arg^9^-BK [30].

## 4. Probing Immunoreactive BK Degradation In Vitro

**Figure 3:**
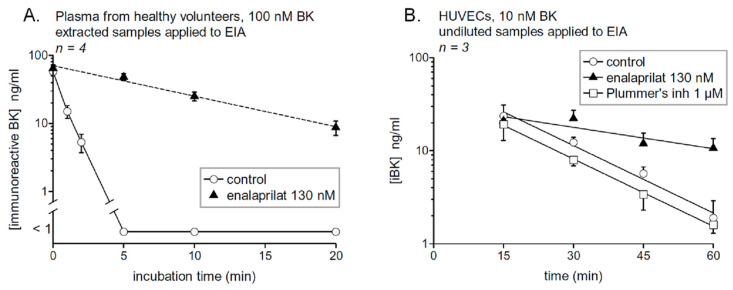
(**A**) Degradation of synthetic BK (100 nM) added to human plasma from healthy subjects in the presence or absence of the ACE inhibitor enalaprilat. Samples were incubated at 37 °C under agitation for the indicated time periods before extraction and EIA determination of BK. The number of replicated from different healthy donors is indicated by “*n*”. In the presence of the ACE inhibitor, the data are compatible with a first order decay with a half-life of 7.1 min, whereas BK was completely cleared in 5 min the absence of enalaprilat (*t*_1/2_ 34 s). Reproduced from [18]. (**B**) Catabolism of BK, measured as [iBK], by intact HUVECs as a function of time. Values are means ± s.e.m. of 3 determinations. A first order decay model has been applied (*t*_1/2_ values reported in main text). BK added to similar wells without cells (nude plastic) was stable when incubated in an identical manner (data not shown).

In vivo assessment of the relative importance of kininases in BK inactivation in rats, based on specific peptidase inhibitors, has shown the major role of ACE and the significant role of aminopeptidase P and neutral endopeptidase, but dipeptidyl peptidase IV was not quantitatively important [17]. Cyr et al. [16] used both BK and des-Arg^9^-BK EIAs to assess the half-life of both synthetic peptides in human serum. The half-life of BK was 27 sec, ACE accounting for most of the clearance activity, as the *t*_1/2_ value increased nine-fold in the presence of the ACE inhibitor enalaprilat. We essentially confirmed these results using the commercial EIA, a lower initial BK concentration and human plasma [18] (Figure 3A). Arginine carboxypeptidases (kininase I), producing des-Arg^9^-BK from BK, was a very minor pathway of metabolism (11%), but more important when ACE was blocked (50%) [16]. The inactivation of des-Arg^9^-BK is also mainly mediated by ACE (*t*_1/2_ doubled in the presence of enalaprilat). Aminopeptidase P is felt to be important in the degradation of kinins, but this cannot be ascertained using the EIAs as the removal of the Arg^1^ residue is not detected by the exploited antibodies.

**Figure 4:**
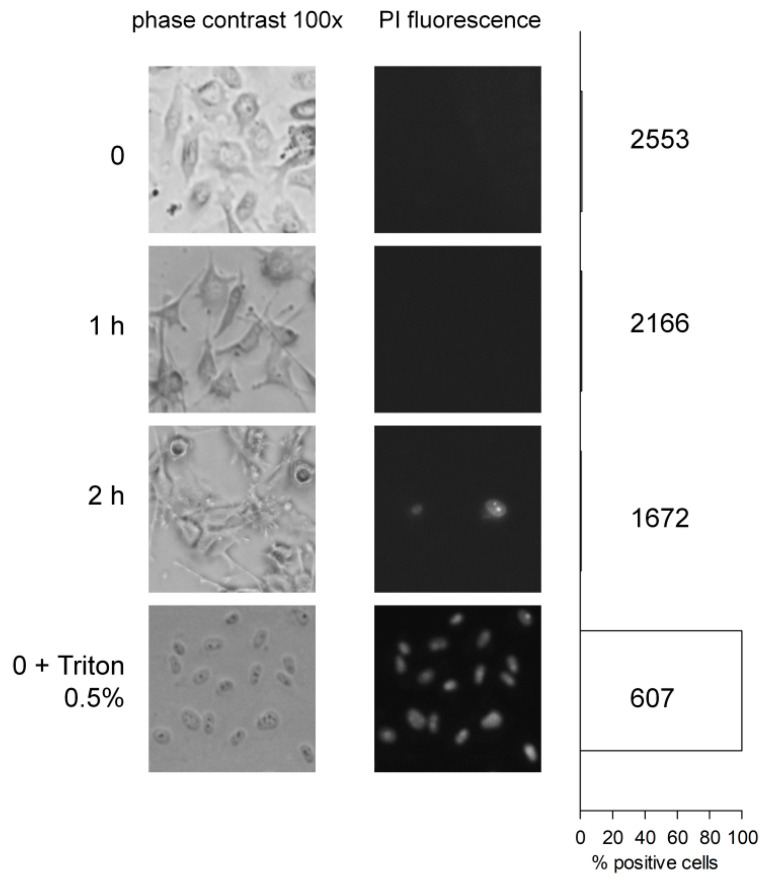
Effect of incubation under agitation in Hank’s balanced salt solution containing BK (500 nM) on adherent HUVECs (matched phase contrast and red fluorescence fields, original magnification 100×). Propidium iodide (PI) staining was performed as a test of viability (positive control, Triton X100, 0.5%). Total counted cell numbers are given next to the histograms.

ACE activity in serum or blood plasma corresponds, in healthy individuals, to a form of the protein shed from the vascular endothelial cells in an apparently regulated manner [34]. ACE, membrane arginine carboxypeptidase (carboxypeptidase M), aminopeptidase P and other potential kininases are expressed at the surface of vascular endothelial cells. Primary cultures of human umbilical vein endothelial cells (HUVECs) are a standard model for which significant information is available. These cells express at their surface a high number of ACE molecule, as judged from the saturable binding of [^3^H]enalaprilat binding (*K*_D_ 0.23 nM) [35] and this binding was displaced by BK and Lys-BK, but much less efficiently by their des-Arg^9^ forms [36]. Based on a radioimmunoassay of BK, adherent HUVECs maintained at 37 °C without serum cleared BK (10 ng/mL) with a half-life of about 50 min. The ACE inhibitor was reported to halt completely the iBK breakdown, despite a minor contribution of neutral endopeptidase [37]. Using the Phoenix Pharmaceuticals EIA, we have verified the degradation of BK (10 nM) by HUVECs (see Materials and Methods for the procedure). The cells, kept under agitation, remained adherent and intact (Figure 4), which is important because lyzed HUVECs degrade BK more rapidly by unknown pathways [37]. iBK was cleared with a *t*_1/2_ of 12.9 min, and this was largely attributable to ACE as enalaprilat increased the *t*_1/2_ value to 47.2 min (Figure 3B). Plummer’s inhibitor, that blocks arginine carboxypeptidases such as carboxypeptidase M, did not exert a significant effect on the system (iBK *t*_1/2_ 12.3 min). The initial recovery of BK was apparently somewhat higher than the added amount of BK in the physiological fluid (10 nM), an artefact possibly due to interference from some proteins released from the cells as the supernatants were directly applied to the EIA without extraction.

Human healthy subjects who took two daily doses of enalapril maleate, the prodrug of enalaprilat, exhibited a ~5-fold increase of BK *t*_1/2_ added ex vivo to their plasma relative to the pre-treatment value [18]. Altogether, the findings illustrate that BK is highly unstable and may contribute to the acquired AE associated with ACE inhibitors if, for some reason, important generation of kinins is triggered. The development of double peptidase inhibitors for ACE and neutral endopeptidase (neprilysin) is still being contemplated as an anti-hypertensive after the failure of omapatrilat, which caused relatively frequent AE attacks [38]. The possible importance of the rapid clearance of BK for patients with HAE is the protection from the continuous production of BK, as judged from HK consumption and kallikrein activity (see below).

Years ago, it has been found that BK can be metabolized by two cycles of ACE catalytic activity, successively producing the fragments BK_1–7_, then BK_1–5;_ they are both inactive on B_1_ or B_2_ receptors [39]. The possible interest of BK_1–5_ is that it is more stable than most kinins and can be detected in clinical samples, using mass spectrometry for instance [40]. Thus, changes in BK_1–5_ plasma concentrations constitute a more discriminative proof of kallikrein–kinin system activation than those of BK in patients undergoing cardiopulmonary bypass, whether or not ACE was pharmacologically blocked [40].

## 5. Profiling Immunoreactive BK Generation In Vitro: Modeling Hereditary Angioedema (HAE)

The main thrust of our investigations based on iBK measurements was the investigation of the physiopathology of different forms of HAE to detect excessive kinin formation. This point of view is different from that of other laboratories that evidenced continuous HK consumption or enzymatic activity of plasma kallikrein in HAE dependent or not on C1-INH deficiency during remission [41,42,43], suggesting an inherently unstable contact system. To establish profiles of iBK formation, citrated blood or plasma obtained from healthy volunteers or HAE patients in remission was incubated at 37 °C under agitation for up to 2 h, alone or in the presence of one of three standardized stimuli (Figure 1): the particulate material Kontact-APTT™ to activate the contact system, human recombinant KLK-1 to probe the alternate kinin generation pathway, and tPA, known to release iBK in vitro with a slow kinetics [20].

**Figure 5:**
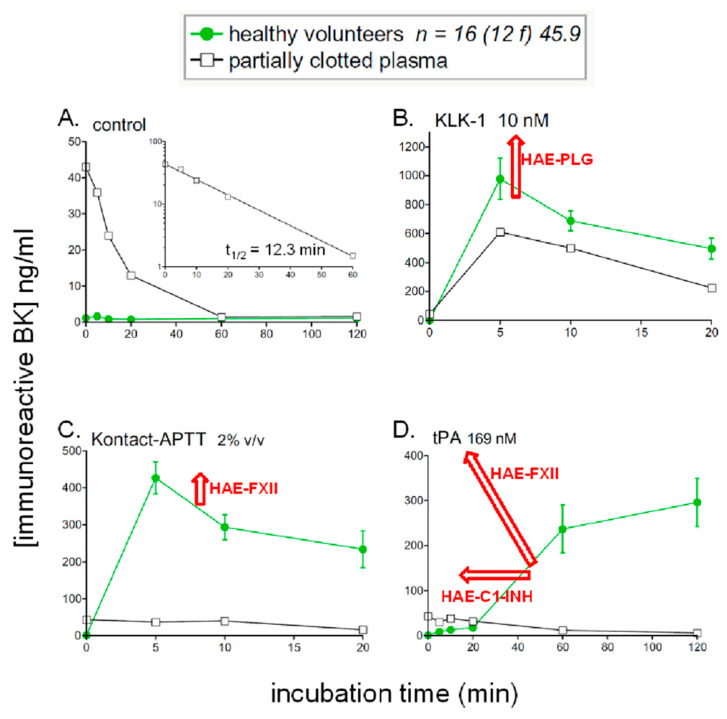
Kinetics of iBK concentrations as a function of time and stimulation in samples of normal plasma incubated at 37 °C in the presence of enalaprilat (130 nM). Panels represent different forms of in vitro stimulation added to plasma at time zero: (**A**) control; (**B**) KLK-1 10 nM; (**C**) Kontact-APTT™ 2% *v*/*v*; (**D**) tPA 169 nM. The data is from the fusion of the 2 control groups (healthy volunteers) from [18,44]. The average age is 45.9 and 12 females are represented in this group of 16 subjects. Indications of significant variations induced by specific HAE subtypes are indicated by red arrows. Measurements made from a partly clotted plasma sample are also shown: in the presence of enalaprilat but no other stimulant, the sizeable concentration of BK at time zero decreases according to a first-order elimination kinetics (panel A, *t*_1/2_ 12.3 min, inset).

A perhaps unexpected result was that the low background of iBK formation observed in control plasma in the absence of stimulant is not modified in patients with HAE-C1-INH, HAE-FXII or HAE-PLG [18,44] (Figure 5A). HAE patients are not symptomatic most of the time: the buffering action of peptidases and protease inhibitors may protect patients from a possible low-grade BK formation during remissions. The plasma or whole blood from HAE patients with C1-INH deficiency showed only one consistent change relative to control iBK formation profiles: in vitro stimulation with tPA considerably accelerated the slow formation of iBK seen in controls [12,44]. The use of specific inhibitors showed that this accelerated response was dependent on the activities of plasmin, FXII and, ultimately, plasma kallikrein. Thus, HAE-C1-INH plasma exhibits a form of hypersensitivity to fibrinolysis which may be related to several of the known triggering factors of attacks, such as mechanical trauma, surgery, infection, menstruation, physical exertion, etc., but also mental stress, the second most frequent triggering factor [45]. Indeed, a whole body of evidence has shown that coagulation and fibrinolysis are significantly activated by mental stress in a healthy subject [46].

HAE-FXII plasma exhibited an even more explosive early response to tPA in vitro [18] (Figure 5), consistent with the very rapid cleavage and activation of the T328K and T328R variants of FXII by plasmin [23]. HAE-FXII essentially affects females in an estrogen-dependent manner and males may carry and transmit the mutation, but are not symptomatic [47]. However, the hormonal status had no obvious influence on the effect of tPA on iBK formation, as the plasma of post-menopausal females have the same profiles [18] and estrogen could rather alter endothelial biology. The plasma of HAE-FXII patients also responds significantly more to the direct contact system activator Kontact-APTT™ in a manner that is not dependant on plasmin [18], suggesting a somewhat increased activity of the variant T328K of FXII variant in the mutual activation reaction with plasma kallikrein.

In their seminal paper about the discovery of the K330E variant of plasminogen, Bork et al. [48] noted that the mutant form of plasmin has a normal catalytic activity, and the idiosyncratic interaction of the mutant plasminogen with another unknown protein was raised as a hypothesis. In agreement with this, the profile of iBK generation under tPA stimulation was not different from that of healthy volunteers [18], although urokinase plasminogen activator has recently been reported to generate increased plasmin activity from the mutant form [49]. The only significantly different iBK profile in HAE-PLG was an increased effect of KLK-1 at the early incubation time 5 min, an unexpected finding suggesting interaction of the mutant plasminogen with KLK-1 (facilitatory) or with its serpin inhibitor kallistatin (inhibitory interaction) [18]. This has to be experimentally confirmed, but may be related to the clinical presentation of HAE-PLG that very often involves the swelling of the lips, tongue and contiguous tissues such as the face and larynx. The possible connection is that KLK-1 is very abundant in the salivary glands and the catalytically active form of this protease is found in human saliva [50]. Thus, the generally overlooked kinin-forming KLK-1 may be responsible, at least in part, for HAE-PLG. It has been recently reported that the administration of C1-INH concentrates has only a variable therapeutic effect in HAE-PLG and that some patients are completely refractory [51], possibly supporting a role for kinin generation outside the contact system in this form of the disease.

One stored plasma sample that was received in our laboratory was partly clotted and excluded from our previous studies. However, submitting the sample to in vitro incubation and stimulation produced revealing results about the functioning of the contact system (Figure 5, overlaid curves). Firstly, the unstimulated sample contained at time zero a sizeable iBK concentration that decreased as a function of time with a *t*_1/2_ of 12.3 min (Figure 5A, inset), marginally superior to the value of 7.1 min for the *t*_1/2_ measured for exogenous BK in the presence of enalaprilat (Figure 3A). Secondly, the partly clotted plasma sample released very little iBK in response to Kontact-APTT™ or tPA (Figure 5C,D), suggesting a major depletion of contact system components. Finally, KLK-1 released important quantities of iBK from the partly clotted sample (Figure 5B), within the range of healthy volunteers (superior to those measured in three of the 16 healthy donors), supporting that KLK-1 and LK are part of a separate kallikrein–kinin system.

## 6. Areas of Uncertainty: Role of BK B_1_ Receptors and Tissue Kallikrein (KLK-1) in Angioedema States

The BK B_1_ receptor is a peculiar G protein-coupled receptor that is strongly regulated at the transcriptional level by tissue injury and cytokines [5]. Its selectivity to kinin metabolite generated by arginine carboxypeptidases has been described above. There has been much speculation about the participation of B_1_ receptors in attacks of AE. Let us note first that the B_1_ receptor is not a receptor for peptides generated by the classical contact system, BK or des-Arg^9^-BK (see above). This has been experimentally verified: the extracts of whole blood stimulated with Kontact-APTT™ contained high levels of iBK that were active on the recombinant B_2_ receptor (cFos signaling), but the extracts were not active on the recombinant B_1_ receptor in replicated and controlled experiments [12]. As mentioned above, the human B_1_ receptor is functionally compartmentalized with tissue kallikrein (KLK-1) that produces Lys-BK and, secondarily, Lys-des-Arg^9^-BK. Thus, B_1_ receptor participation in HAE forms driven by the contact system may be less important than what several authors have described.

An elaborate in vitro simulation of the effect of HAE-C1-INH plasma on human endothelial cells (HUVECs) has been reported [52]. A kinin content in HAE-C1-INH plasma obtained during attacks has been postulated on the basis of an endothelial cell permeability assay based on a Transwell model: the plasma permeabilizing effect was partially inhibited by B_2_ receptor or B_1_ receptor antagonists, and was totally prevented by the mixture of the two antagonists. As opposed to these observations, (1) the expression of B_1_ receptors by vascular cells cultured on plastic in serum is not surprising and is largely an artefact (for HUVECs notably) [53]; (2) what was transferred from patient’s plasma to wells to activate B_1_ receptors? Not necessarily peptides. Perhaps secondarily released KLK-1, explaining a stimulation of the B_1_ receptors? Some other protease? Exquisitely specific biotechnological inhibitors for several proteases now exist to address such questions. Thus, a parsimony principle may be invoked to state that there is no proof on B_1_ receptor participation in attacks of HAE-C1-INH, until proper clinical evidence is collected.

We described above the intriguing observation about KLK-1 potentiation in HAE-PLG plasma. The highly efficient KLK-1/LK kallikrein–kinin system (Figure 1 and Figure 3B) has been overlooked in AE states and may be worth considering. KLK-1 is highly concentrated in the salivary glands, and the oral/facial manifestations of AE are frequent in HAE-PLG, but also in the acquired AE state associated with ACE inhibitors [19]. KLK-1 mediation has the potential to include the B_1_ receptor in the mediation of vascular effects, as discussed above. The efficacy of icatibant, a selective B_2_ receptor antagonist, is controversial in ACE inhibitor-induced AE [54], perhaps in line with this. Although synthetic des-Arg^10^-icatibant is a potent B_1_ receptor antagonist [5], it is not a spontaneous metabolite of icatibant due to the non-natural residues in position 7 and 8 (D-Tic^7^, Oic^8^) that preclude the binding to arginine-carboxypeptidases. The in vivo metabolites of icatibant are well known and represent fragments 1–5 and 7–10 of this decapeptide [55].

## 7. Conclusions

The systematic study of BK generation and clearance is an original point of view to investigate the “weak spots” that may trigger attacks of BK-mediated AE states, the natural detoxifying action of kininases being considerable. Inhibition of such a clearance mechanism is likely involved in the acquired AE associated with ACE inhibitors. In these investigations, we stayed as close as possible to the in vivo condition where protease inhibitors abound. All our HAE patients were seen in remission in order to isolate excessive responses of the kallikrein–kinin system to one of the standardized stimuli. HAE-C1-INH and HAE-FXII plasmas are both hyperresponsive to fibrinolysis activation. Other intriguing observations suggest a possible role of the alternate KLK-1/LK kallikrein–kinin system in specific forms of AE. However, the present approach says little about important local endothelial factors that may modulate HAE states.

## 8. Materials and Methods (for Original Results)

### 8.1. Degradation of BK by Human Endothelial Cells

The local ethics committee (CHU de Québec-Université Laval) had approved the anonymous use of human umbilical cord segments obtained after normal deliveries (File no. 2017-3720). Primary cultures of endothelial cells from the human umbilical vein (HUVECs) were obtained and propagated as described [53]. These cells were characterized in immunofluorescence experiments by their morphology and expression of von Willebrand factor using primary antibodies from Dako (polyclonals) and the appropriate fluorophore-conjugated antibodies.

HUVECs were cultured until confluency in 6-well plates; the culture medium was then removed, rinsed and refilled (3 mL/well) with preheated (37 °C) Hank’s balanced salt solution (pH 7.4). Synthetic BK (final concentration 10 nM) was added, optionally with an enzyme inhibitor, and the plates were incubated for up to 60 min in a warmed chamber (37 °C) under continuous oscillating agitation. The cells remained viable for 2 h (see below). Control plates without cells (nude plastic) were used as controls. After the incubation, 300 µL fluid from each well was removed, added to 1.5 mL ice-cold ethanol and the mixture was further incubated on ice for 1 h. At the end, each tube was centrifuged and the supernatants set aside in a new set of tube submitted to complete evaporation in a SpeedVac apparatus and preserved at −80 °C. The dried residues were later resuspended in 300 µL with the supplied enzyme immunoassay (EIA) buffer; undiluted 50 µL samples were directly applied in duplicate to the BK EIA as recommended by the manufacturer (Phoenix Pharmaceuticals, Burlingame, CA; cat. no. EK-009-01; 96-well plate format).

In addition, a microscopic study of the morphology of HUVECs maintained in Hank’s balanced salt solution containing 500 nM BK under agitation for 0–2 h was conducted. The study included a staining with propidium iodide (2.5 µM, 4 min, room temperature) as a further test for endothelial cell viability under the conditions applied to measure BK breakdown. The proportion of propidium iodide stained nucleus (red fluorescence) was calculated from a large photographic record (100×). Cells acutely treated with Triton X-100 (0.5%) were positive controls.

### 8.2. Generation and Degradation of BK by Human Plasma in Health and Disease

The local ethical review board (Comité d’éthique de la recherche, CHU de Québec-Université Laval) granted ethical approval to carry out the study involving blood donations from adult healthy volunteers and HAE patients 16 years old or older (files no. 2018-3857, 2020-4696). Citrated plasma was obtained, processed, and extracted precisely as described [18,44] for the determination of synthetic BK degradation and iBK generation under standardized in vitro stimulation. The same EIA was applied to plasma extracts (1:100 or 1:1000) diluted with the assay buffer.

## Figures and Tables

**Table 1:** List of abbreviations.

Abbreviation	Standing for
ACE	angiotensin-I converting enzyme
AE	angioedema
APTT	activated partial thromboplastin time
B_1_R	bradykinin B_1_ receptor
B_2_R	bradykinin B_2_ receptor
BK	bradykinin
C1-INH	C1-esterase inhibitor
c-Fos	a transcription factor
EIA	enzyme immunoassay
ERK1/2	extracellular signal-regulated kinases 1 and 2
*F12*	gene encoding factor XII
FDA	U.S. Food and Drug Administration
FXI	coagulation factor XI
FXII	coagulation factor XII
HAE	hereditary angioedema
HAE-C1-INH	HAE caused by *SERPING1* variants
HAE-FXII	HAE caused by *F12* variants
HAE-PLG	HAE caused a *PLG* variant
HK	high molecular weight kininogen
HPLC	high-performance liquid chromatography
HUVEC	human umbilical vein endothelial cell
iBK	immunoreactive bradykinin
K_D_	binding dissociation constant
KLK-1	tissue kallikrein
*KNG1*	gene encoding kininogens
Kontact-APTT™	a commcercial reagent that activates the contact system
LK	low molecular weight kininogen
Lys-BK	lysyl-bradykinin, kallidin
PI	propidium iodide
*PLG*	gene encoding plasminogen
s.e.m.	standard error of the mean
*t*_1/2_	half-life
tPA	tissue plasminogen activator
VE-cadherin	vascular endothelial cadherin
